# Association of Painful Musculoskeletal Conditions and Migraine Headache With Mental and Sleep Disorders Among Adults With Disabilities, Spain, 2007–2008

**DOI:** 10.5888/pcd11.130144

**Published:** 2014-02-27

**Authors:** Alejandro Salazar, María Dueñas, Begoña Ojeda, Inmaculada Failde

**Affiliations:** Author Affiliations: María Dueñas, Begoña Ojeda, Inmaculada Failde, Preventive Medicine and Public Health Area, University of Cádiz, Cádiz, Spain.

## Abstract

**Introduction:**

The aim of this study was to determine the prevalence of painful musculoskeletal conditions and migraine headache or any other headache in a sample of Spanish adults with disabilities and their association with anxiety, depression, and sleep disorders.

**Methods:**

This cross-sectional study analyzed data from the Spanish national disability and dependence survey (2007–2008) of 16,932 adults aged 18 or older who have disabilities. The prevalence (95% confidence interval [CI]) of painful musculoskeletal conditions was determined according to a diagnosis of arthritis, osteoarthritis, rheumatoid arthritis, ankylosing spondylitis, muscular dystrophy, and neck or back pain. The prevalence of migraine or other headache was also calculated. Factors associated with these painful conditions were analyzed separately for men and women by using a logistic regression model.

**Results:**

The prevalence of painful musculoskeletal conditions was 66.9% (95% CI, 66.2%–67.6%) and that of migraine or other headache was 23.4% (95% CI, 22.8%–24.1%), both of which were higher in women than in men. Factors associated with these conditions in both men and women included older age, a sleep disorder, and concomitant chronic anxiety and/or depression.

**Conclusion:**

The prevalence of painful musculoskeletal conditions and migraine or other headache is high in people with disability in Spain, especially in women, and these conditions often coexist with depression, anxiety, and/or a sleep disorder. To design programs for rehabilitating and improving the quality of life of adults with disability and painful conditions, treatments for mental and/or sleep disorders should be considered in addition to conventional treatments.

## MEDSCAPE CME

Medscape, LLC is pleased to provide online continuing medical education (CME) for this journal article, allowing clinicians the opportunity to earn CME credit.

This activity has been planned and implemented in accordance with the Essential Areas and policies of the Accreditation Council for Continuing Medical Education through the joint sponsorship of Medscape, LLC and Preventing Chronic Disease. Medscape, LLC is accredited by the ACCME to provide continuing medical education for physicians.

Medscape, LLC designates this Journal-based CME activity for a maximum of 1 **AMA PRA Category 1 Credit(s)™**. Physicians should claim only the credit commensurate with the extent of their participation in the activity.

All other clinicians completing this activity will be issued a certificate of participation. To participate in this journal CME activity: (1) review the learning objectives and author disclosures; (2) study the education content; (3) take the post-test with a 70% minimum passing score and complete the evaluation at www.medscape.org/journal/pcd (4) view/print certificate.

**Release date: Febuary 27, 2014; Expiration date: February 27, 2014**

### Learning Objectives

Upon completion of this activity, participants will be able to:

- Distinguish the most common cause of physical disability in Spain
- Analyze the prevalence of chronic pain among adults with disability
- Assess epidemiologic trends in chronic pain among adults with disability
- Evaluate risk factors for chronic pain among adults with disability

**EDITORS**

Ellen Taratus, editor, *Preventing Chronic Disease*. Disclosure: Ellen Taratus has disclosed no relevant financial relationships.

**CME AUTHOR**

Charles P. Vega, MD, Associate Professor and Residency Director, Department of Family Medicine, University of California, Irvine. Disclosure: Charles P. Vega, MD, has disclosed no relevant financial relationships.

**AUTHORS AND CREDENTIALS**

Disclosures: Alejandro Salazar, María Dueñas, Begoña Ojeda, and Inmaculada Failde have disclosed no relevant financial relationships.

Affiliations: Alejandro Salazar, MSc, María Dueñas, PhD, Begoña Ojeda, MSc, and Inmaculada Failde, MD, PhD, Preventive Medicine and Public Health Area, University of Cádiz, Cádiz, Spain.

## Introduction

Chronic pain is a common condition that affects one-third of the population in Europe and 12% of the Spanish population, accounting for a large number of medical consultations and a significant proportion of health care costs (1). Chronic pain is linked to several pathologies, such as rheumatic diseases (2), although other conditions, such as neck or back pain and migraine or other headaches, are common causes of chronic pain in the general population (3,4).

Disability is also a prevalent condition, affecting 8.9% of the Spanish population (5), and chronic pain is a common complaint in such patients (6). Osteoarthritis represents the main cause of physical disability (7) and chronic pain (1) in Spain. However, to our knowledge, the prevalence of other diseases that cause chronic pain, such as neck or back pain and migraine or other headache, has not been analyzed in people with disability. Thompson et al (8) emphasize the need to understand the factors that are related to pain in people with disability to find alternative treatments for reducing their levels of disability.

Evidence suggests that anxiety and depression are associated with increased pain sensitivity and pain-related disability (9), comorbid states that are more disabling than either condition alone (10). Likewise, sleep disorders have also been linked with chronic pain, and people who experience pain-related sleep disturbances are significantly more disabled than those who do not (11).

The objective of this study was to investigate the prevalence of painful musculoskeletal conditions and migraine or other headache and their link with anxiety, depression, and sleep disorders in a sample of Spanish adults with disability.

## Methods

### Study sample and variables

In this cross-sectional study, we used secondary data from the last national disability and dependence survey conducted in Spain from November 2007 through February 2008 by the National Institute of Statistics. Survey details are available elsewhere (12). A representative sample of 96,073 households was selected randomly from the Spanish population (response rate, 95.6%), and all residents older than 6 years with some form of disability, defined as “any important limitation in performing activities of daily living that has lasted, or is expected to last longer than 1 year, and that has its origin in a deficiency” were included in the survey. Individuals who had overcome their disability by means of external technical assistance or supervised assistance were also included.

On the basis of the International Classification of Functioning, Disability and Health (13), we considered the following categories or types of disabilities: vision, hearing, communication, learning and application of knowledge and task performance (only cognitive or intellectual problems), mobility, self-care, domestic life, and interactions and relationships.

We examined the following chronic conditions, in which pain is a key symptom and forms part of the diagnosis, and organized them into 4 groups. Group 1 consisted of arthritis, osteoarthritis, rheumatoid arthritis, and ankylosing spondylitis (rheumatic diseases); group 2, muscular dystrophy; group 3, neck or back pain; group 4, migraine or other headache. Groups 1, 2, and 3 constituted the single category of painful musculoskeletal conditions for this study. We collected information on these conditions directly from the survey; in the survey, participants indicated whether they had been diagnosed with any of these conditions by a physician. One question was used to ask about all conditions in a group, so we could not obtain information for each condition. We also collected information on the following factors: sex; age; marital status; highest level of education attained; the number of hours of sleep per day (categorized as ≤6 hours and >6 hours [14]); the presence of chronic anxiety and/or chronic depression diagnosed by a physician and recorded in the survey; and the financial benefits or compensations received in the previous 12 months as a result of the disability. We defined a sleep disorder as sleeping 6 hours or less per day.

### Statistical analysis

We calculated the prevalence and 95% confidence intervals (CIs) of each of the 4 groups of conditions, the group of painful musculoskeletal conditions, and all conditions combined. We also calculated the prevalence by sex, age category, marital status, level of education attained, hours of sleep per day, presence of anxiety, presence of depression, and financial benefit or compensation received in the previous 12 months as a result of the disability. These analyses were reproduced in men and women separately.

To determine the factors associated with the presence of painful conditions, we performed 2 logistic regression models (one for men and another for women) in which the dependent variable was the presence or absence of the painful condition and the independent variables were all the other variables included in the study. We used the Hosmer–Lemeshow test to measure the goodness-of-fit of the model and the statistical software SPSS version17.0 (IBM Corp, Armonk, New York) to perform the analyses. The level of significance was established at *P* < .05.

## Results

### Characteristics of the study sample

Of the 16,932 adults with disability included in the study, 64.0% were women. The average age was 68.5 (standard deviation [SD], 16.3 y), and 64.5% of participants were 65 or older. In addition, 53.4% were married, 66.6% had complete or incomplete primary education, and 11.5% were illiterate. Of the participants, 15.7% had been diagnosed with chronic anxiety and 21.3% with chronic depression; 31.6% slept 6 hours or less per day, and 8.7% had received financial benefit or compensation in the previous 12 months as a result of their disability (Table 1).

**Table 1:** Characteristics of a Sample of Adults With Disabilities, Study on Painful Musculoskeletal Conditions^a^ and Mental and Sleep Disorders, Spain, 2007–2008^b^

Variable^c^	All Adults With Disabilities	Adults With Painful Conditions	Men With Painful Conditions	Women With Painful Conditions	*P *Value^d^
**Sex, n**	16,932	13,032	3,929	9,103	—
Female	10,842 (64.0)	9,103 (69.9)	—	—	—
**Age category, n**	16,932	13,032	3,929	9,103	—
18–44 y	1,645 (9.7)	1,012 (7.8)	406 (10.3)	606 (6.7)	<.001
45–64 y	4,366 (25.8)	3,337 (25.6)	1,130 (28.8)	2,207 (24.3)	
65–74 y	3,438 (20.3)	2,735 (21.0)	821 (20.9)	1,914 (21.0)	
75–84 y	5,015 (29.6)	4,002 (30.7)	1,121 (28.5)	2,881 (31.6)	
≥85 y	2,468 (14.6)	1,946 (14.9)	451 (11.5)	1,495 (16.4)	
**Marital status, n**	16,930	13,031	3,929	9,102	—
Single	2,298 (13.6)	1,393 (10.7)	549 (14.0)	844 (9.2)	<.001
Married	9,042 (53.4)	7,003 (53.7)	2,751 (70.0)	4,252 (46.7)	
Widow(er)	4,901 (28.9)	4,081 (31.3)	471 (12.0)	3,610 (39.7)	
Separated/divorced	689 (4.1)	554 (4.3)	158 (4.0)	396 (4.4)	
**Level of education attained, n**	16,914	13,019	3,927	9,092	—
Illiterate	1,942 (11.5)	1,523 (11.7)	277 (7.1)	1,246 (13.7)	<.001
Complete or incomplete primary education	11,266 (66.6)	8,768 (67.3)	2,608 (66.4)	6,160 (67.8)	
Complete secondary education	2,734 (16.1)	2,027 (15.6)	756 (19.2)	1,271 (14.0)	
Complete higher education	972 (5.8)	701 (5.4)	286 (7.3)	415 (4.5)	
**Hours of sleep per day,^e^ n**	16,536	12,824	3,886	8,938	—
≤6	5,218 (31.6)	4,454 (34.7)	1,096 (28.2)	3,358 (37.6)	<.001
>6	11,318 (68.4)	8,370 (65.3)	2,790 (71.8)	5,580 (62.4)	
**Chronic anxiety, n**	16,787	12,992	3,914	9,078	—
Yes	2,642 (15.7)	2,388 (18.4)	539 (13.8)	1,849 (20.4)	<.001
**Chronic depression, n**	16,788	12,993	3,914	9,079	—
Yes	3,572 (21.3)	3,139 (24.2)	675 (17.2)	2,464 (27.1)	<.001
**Financial benefit received in the previous 12 months as a result of the disability, n**	16,852	13,015	3,923	9,092	—
Yes	1,471 (8.7)	1,015 (7.8)	407 (10.4)	608 (6.7)	<.001

Abbreviation: —, not applicable.

a Painful musculoskeletal conditions considered were arthritis, osteoarthritis, rheumatoid arthritis, and ankylosing spondylitis (rheumatic diseases); muscular dystrophy; neck or back pain; migraine or other headache.

b Values are numbers (percentages) unless otherwise indicated. Source of data: Spanish national disability and dependence survey (12).

c The *n* after each variable indicates the number of survey participants who answered question.

d χ^2^ test for the difference between men and women.

e Presence of a sleep disorder was defined as 6 hours or less of sleep per day.

Among the participants with a painful condition, 69.9% were women, and 66.6% were 65 or older. In addition, 53.7% were married, 67.3% had complete or incomplete primary education, 34.7% slept 6 or less hours per day, 18.4% had been diagnosed with chronic anxiety and 24.2% with chronic depression, and 7.8% had received financial benefit or compensation in the previous 12 months as a result of their disability (Table 1).

In the analysis of characteristics by sex, we observed several differences. In general, women were older than men and had a lower educational level; the proportion of widows was higher than that of widowers; women slept fewer hours, more women had chronic anxiety and chronic depression, and women received less financial compensation, especially contributory pensions, as a result of their disability (Table 1).

### Prevalence of painful conditions and associated factors

The overall prevalence of painful conditions was 77.0% (95% CI, 76.3%–77.6%). The prevalence of painful musculoskeletal conditions was 66.9% (95% CI, 66.2%–67.6%), and the prevalence of migraine or other headache was 23.4% (95% CI, 22.8%–24.1%). Among the painful musculoskeletal conditions, group 1 was the most prevalent (62.1%; 95% CI, 61.3%–62.8%) followed by group 3 (51.3%; 95% CI, 50.5%–52.0%) (Figure). The overall prevalence of painful conditions was higher in women than in men, in widows and widowers than in other categories of marital status, in participants with anxiety or depression than in those who did not have those conditions, in participants who slept 6 hours or less, and in those who did not receive any financial compensation as a result of their disability. In addition, the prevalence of painful conditions increased with age, from 61.5% in participants aged 18 to 44 to approximately 79% in the older age groups (≥65), and it decreased as the level of education increased. We observed an analogous situation in the analysis by sex, although women had a higher prevalence than men in all analyses (Table 2).

**Figure:**
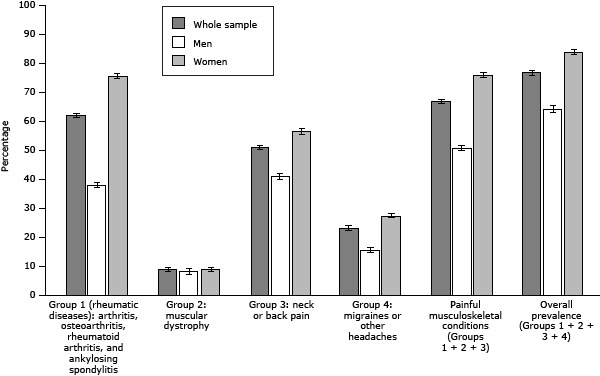
Prevalence of painful conditions in Spanish adults with disabilities, by group of conditions and by sex. National disability and dependence survey, Spain, 2007–2008. Differences between men and women were significant at *P* <.001 (determined by χ^2^ test) for all groups of conditions, except muscular dystrophy (*P* = .21). Abbreviation: CI, confidence interval. **Table:** 

Group of conditions	All, Prevalence (95% CI)	Men, Prevalence (95% CI)	Women, Prevalence (95% CI)	*P* Value^a^
Group 1: arthritis, osteoarthritis, rheumatoid arthritis, and ankylosing spondylitis (rheumatic diseases)	62.1 (61.3–62.8)	38.1 (37.2–39.1)	75.8 (74.9–76.5)	<.001
Group 2: muscular dystrophy	9.1 (8.7–9.5)	8.6 (8.0–9.4)	9.3 (8.8–9.9)	.21
Group 3: neck or back pain	51.3 (50.5–52.0)	41.2 (39.9–42.4)	56.9 (56.0–57.9)	<.001
Group 4: migraine or other headache	23.4 (22.8–24.1)	15.9 (15.0–16.8)	27.7 (26.8–28.5)	<.001
Painful musculoskeletal conditions (groups 1, 2, and 3)	66.9 (66.2–67.6)	51.0 (50.1–52.0)	76.1 (75.3–76.8)	<.001
Overall prevalence (all groups)	77.0 (76.3–77.6)	64.5 (63.3–65.7)	84.0 (83.3–84.7)	<.001

Abbreviation: CI, confidence interval.

^a^ Determined by χ^2^ test.

**Table 2:** Prevalence of Painful Musculoskeletal Conditions^a^ by Other Variables in a Sample of Adults With Disabilities, Study on Painful Musculoskeletal Conditions and Mental and Sleep Disorders, Spain, 2007–2008^b^

Variable	Prevalence, % (95% CI)			*P* Value^c^
	All Adults	Men	Women	
**Age category, y**				
18–44	61.5 (59.1–63.9)	51.9 (48.3–55.4)	70.3 (67.2–73.4)	<.001
45–64	76.4 (75.2–77.7)	64.5 (62.2–66.8)	84.4 (83.0–85.8)	<.001
65–74	79.6 (78.2–80.9)	65.6 (62.9–68.3)	87.6 (86.2–89.0)	<.001
75–84	79.8 (78.7–80.9)	68.0 (65.7–70.3)	85.6 (84.4–86.8)	<.001
≥85	78.9 (77.2–80.5)	69.0 (65.3–72.6)	82.4 (80.6–84.2)	<.001
**Marital status**				
Single	60.6 (58.6–62.6)	49.3 (46.3–52.3)	71.3 (68.7–73.9)	<.001
Married	77.5 (76.6–78.3)	67.3 (65.8–68.7)	85.9 (84.9–86.9)	<.001
Widow(er)	83.3 (82.2–84.3)	70.9 (67.4–74.5)	85.2 (84.1–86.3)	<.001
Separated/divorced	80.4 (77.4–83.4)	71.2 (65.0–77.4)	84.8 (81.4–88.2)	<.001
**Level of education attained**				
Illiterate	78.4 (76.6–80.3)	60.9 (56.3–65.5)	83.8 (81.9–85.7)	<.001
Complete or incomplete primary education	77.8 (77.1–78.6)	65.6 (64.1–67.1)	84.5 (83.7–85.4)	<.001
Complete secondary education	74.1 (72.5–75.8)	63.4 (60.7–66.2)	82.4 (80.5–84.4)	<.001
Complete higher education	72.1 (69.3–75.0)	62.0 (57.5–66.6)	81.2 (77.7–84.7)	<.001
**Hours of sleep per day^d^**				
≤6	85.4 (84.4–86.3)	73.7 (71.4–75.9)	90.0 (89.1–91.0)	<.001
>6	74.0 (73.1–74.8)	62.4 (60.9–63.8)	81.5 (80.6–82.5)	<.001
**Chronic anxiety**				
Yes	90.4 (89.2–91.5)	79.4 (76.3–82.5)	94.2 (93.1–95.3)	<.001
No	75.0 (74.3–75.7)	63.0 (61.7–64.3)	82.2 (81.4–83.1)	<.001
**Chronic depression**				
Yes	87.9 (86.8–89.0)	75.4 (72.5–78.3)	92.0 (91.0–93.1)	<.001
No	74.6 (73.8–75.3)	63.0 (61.7–64.4)	81.9 (81.0–82.7)	<.001
**Financial benefit received in the previous 12 months as a result of the disability**				
Yes	69.0 (66.6–71.4)	60.0 (56.3–63.8)	76.7 (73.7–79.7)	<.001
No	78.0 (77.4–78.7)	65.3 (64.1–66.6)	84.8 (84.1–85.6)	<.001

Abbreviation: CI, confidence interval.

a Painful musculoskeletal conditions considered were arthritis, osteoarthritis, rheumatoid arthritis, and ankylosing spondylitis (rheumatic diseases); muscular dystrophy; neck or back pain; migraine or other headache.

b Source of data: Spanish national disability and dependence survey (12).

c χ^2^ test for the differences between men and women.

d Presence of a sleep disorder was defined as 6 hours or less of sleep per day.

Being older, sleeping 6 hours or fewer per day, and having chronic anxiety or chronic depression were the factors associated with a risk of having chronic pain in men and women. Although we found no evidence to show that associations were stronger in women, all pain-associated factors were more common among women than among men (Table 3).

**Table 3:** Factors Associated With the Presence of Painful Conditions^a^ in a Sample of Men and Women With Disabilities, Study on Painful Musculoskeletal Conditions and Mental and Sleep Disorders, Spain, 2007–2008^b^

Variable	Presence of Painful Conditions in Men^c^ (n = 5,940)			Presence of Painful Conditions in Women^d^ (n = 10,549)		
	Β	OR (CI 95%)	*P* Value^e^	Β	OR (CI 95%)	*P* Value^e^
**Age category, y**						
18–44	1 [Reference]		—	1 [Reference]		—
45–64	0.55	1.73 (1.45–2.07)	<.001	0.79	2.20 (1.81–2.66)	<.001
65–74	0.69	2.00 (1.65–2.42)	<.001	1.20	3.31 (2.69–4.07)	<.001
75–84	0.81	2.25 (1.87–2.69)	<.001	1.10	3.00 (2.49–3.61)	<.001
≥85	0.86	2.37 (1.89–2.97)	<.001	0.91	2.47 (2.02–3.02)	<.001
**Hours of sleep per day^f^**						
≤6	0.46	1.59 (1.39–1.81)	<.001	0.61	1.83 (1.62–2.08)	<.001
>6	1 [Reference]		—	1 [Reference]		—
**Chronic anxiety**						
Yes	0.69	1.99 (1.59–2.50)	<.001	1.00	2.72 (2.19–3.39)	<.001
No	1 [Reference]		—	1 [Reference]		—
**Chronic depression**						
Yes	0.35	1.42 (1.18–1.72)	<.001	0.56	1.74 (1.47–2.07)	<.001
No	1 [Reference]		—	1 [Reference]		—

Abbreviations: OR, odds ratio; CI, confidence interval; —, not applicable; *df*, degrees of freedom.

a Painful musculoskeletal conditions considered were arthritis, osteoarthritis, rheumatoid arthritis, and ankylosing spondylitis (rheumatic diseases); muscular dystrophy; neck or back pain; migraine or other headache.

b Source of data: Spanish national disability and dependence survey (12).

c Hosmer–Lemeshow χ^2^ = 2.8, *df* = 6; *P* = .84.

d Hosmer–Lemeshow χ^2^ = 7.5, *df* = 7; *P* = .38.

e Determined by Wald test.

f Presence of a sleep disorder was defined as 6 hours or less of sleep per day.

## Discussion

Although some studies have analyzed the prevalence of musculoskeletal pain and migraine or other headache among the general Spanish population, this is the first study, to the best of our knowledge, to investigate the prevalence of these painful conditions and their association with anxiety, depression, and sleep disorders in a population of adults with disability in Spain. Improving the knowledge of pain and its related factors in this population could contribute to finding alternative treatments for reducing levels of disability and improving quality of life.

A key finding in our study was the high prevalence of painful conditions among people with disabilities, especially rheumatic diseases. We found especially high rates of painful conditions among women aged 65 or older, those diagnosed with anxiety or depression, and those who slept 6 hours or less per day. We also observed a high prevalence of neck and back pain and migraine or other headache, a phenomenon not described previously in a population with disability.

The prevalences found in our study are consistent with but higher than the prevalences found in populations without disability in Spain. In this latter population, the 1-year prevalence of neck pain and lower back pain is approximately 20% (3), compared with 51% in our study, and the prevalence of migraine or other headache is 11% (4), compared with 23% in our study. Our findings also agree with those of a study that demonstrated that people with disabilities report a higher prevalence of pain, depression, anxiety, and worse quality of life (15). The higher prevalence among women and among the older age groups is consistent with the prevalence among the general population and can be partially explained by the longer life expectancy of women and the increased frequency of conditions that potentially cause pain in older populations (7,16). In a study assessing sex differences in pain-related disability among patients with musculoskeletal pain, women reported a greater intensity of pain and pain-related disability than men, even after controlling for depression, anxiety, and other psychological variables such as self-efficacy (17). Biological and psychosocial influences may predispose men and women to use different coping strategies and cause women to catastrophize more than men (18). A review of sex, gender, and pain described differences in clinical and experimental pain responses between men and women and discussed multiple mechanisms that may contribute to these differences, including gonadal hormones, endogenous pain modulating systems, gender roles, and cognitive/affective factors (19). Despite these considerations, contradictory findings suggest that disability is more directly related to pain in men than in women (20). Such inconclusive results support the need for further research into the sex and gender differences in people with pain and disability to foster future interventions to reduce disparities.

The association described here between painful conditions and anxiety or depression has been addressed in clinical settings and population-based studies (21,22). In these populations, negative emotional factors play an important role in the perception and experience of pain, and pain intensity is consistently documented as a predictor of physical disability and depression (23). The relationship between chronic pain and depression or anxiety is complex, and according to some researchers, reciprocal (24). However, the mechanisms that associate depression and anxiety with increased sensitivity to pain and greater disability remain poorly understood (9). Our group recently demonstrated the responsibility of the locus coeruleus in the onset of depression and anxiety as a consequence of chronic pain (25).

A synergistic effect of mental–physical comorbidity on severe disability has been reported (26), and mechanisms for this synergy have been proposed, including the possibility that depression exacerbates the disabling effect of a chronic physical condition by influencing treatment adherence and healthy behavior (27). Depression can also interfere with the psychological capacity to adjust to physical conditions, and it can affect the perception and appraisal of pain and the ability to cope with it (28).

The stronger link between depression and disability (both physical and mental) in women than in men further complicates the aforementioned relationships, suggesting that depression may have a greater disabling effect on women who suffer from pain (9). Anxiety has also been proposed to potentially mediate the sex differences in pain sensitivity; women tend to report higher levels of anxiety and are more likely to have anxiety disorders. However, increasing evidence suggests that anxiety is more strongly associated with pain responses in men than in women (29), and inconsistent or contradictory results have been reported on the direction of the link between anxiety and chronic pain within sexes and across outcome measures (18). In our study, the association between anxiety and painful conditions was independent of sex, unlike depression.

The link between sleep disorders and painful conditions described in our study is consistent with results reported for people who have pain but no disability; several authors suggest that the link between sleep disturbances and painful conditions is reciprocal (30). In the general population, chronic pain is associated with more symptoms of insomnia, more severe daytime consequences, and more chronic insomnia, thereby playing a major role in the chronicity of insomnia (11).

Our study has several limitations. The main limitation is the use of secondary data obtained for other purposes. The data on rheumatic diseases were not collected individually, and thus disaggregated analysis could not be performed. Also, because of missing values, we were unable to analyze data according to the survey participant’s exact type of disability. Despite these limitations, our study sheds light on the factors associated with the most common painful conditions found in people with disabilities, which to date has received little attention from researchers in Spain. 

Our study also had strengths. A key strength is the large sample size and the inclusion of a group of people who have a disability but no pain, who are not typically included in studies. The inclusion of this group provides a broader perspective on the factors related to painful musculoskeletal conditions and migraine or other headache in people who have a disability. Moreover, the representativeness of the sample and the high response rate imply that the results are extendible to all people with disabilities in Spain. 

Our study demonstrated that the prevalence of certain painful conditions — rheumatic diseases, neck and back pain, and migraine or other headache — is higher in people who have disabilities and that these conditions often co-exist with depression, anxiety, and sleep disorders. These findings suggest that pain-related conditions and their associated disorders should be considered in the design of pain-management programs to rehabilitate patients and improve their quality of life. Our study also reinforces from a translational perspective preclinical data that demonstrate a relationship between chronic pain and mental status.

## Figures and Tables

**Table:** 

**1. The activity supported the learning objectives.**				
**Strongly Disagree**				**Strongly Agree**
1	2	3	4	5
**2. The material was organized clearly for learning to occur.**				
**Strongly Disagree**				**Strongly Agree**
1	2	3	4	5
**3. The content learned from this activity will impact my practice.**				
**Strongly Disagree**				**Strongly Agree**
1	2	3	4	5
**4. The activity was presented objectively and free of commercial bias.**				
**Strongly Disagree**				**Strongly Agree**
1	2	3	4	5

## Post-Test Information

To obtain credit, you should first read the journal article. After reading the article, you should be able to answer the following, related, multiple-choice questions. To complete the questions (with a minimum 70% passing score) and earn continuing medical education (CME) credit, please go to http://www.medscape.org/journal/pcd. Credit cannot be obtained for tests completed on paper, although you may use the worksheet below to keep a record of your answers. You must be a registered user on Medscape.org. If you are not registered on Medscape.org, please click on the "Register" link on the right hand side of the website to register. Only one answer is correct for each question. Once you successfully answer all post-test questions you will be able to view and/or print your certificate. For questions regarding the content of this activity, contact the accredited provider, CME@medscape.net. For technical assistance, contact CME@webmd.net. American Medical Association's Physician's Recognition Award (AMA PRA) credits are accepted in the US as evidence of participation in CME activities. For further information on this award, please refer to http://www.ama-assn.org/ama/pub/about-ama/awards/ama-physicians-recognition-award.page. The AMA has determined that physicians not licensed in the US who participate in this CME activity are eligible for **AMA PRA Category 1 Credits™**. Through agreements that the AMA has made with agencies in some countries, AMA PRA credit may be acceptable as evidence of participation in CME activities. If you are not licensed in the US, please complete the questions online, print the AMA PRA CME credit certificate and present it to your national medical association for review.

## Post-Test Questions

### Article Title: Prevalence of Chronic Conditions Among Medicare Part A Beneficiaries in 2008 and 2010: Are Medicare Beneficiaries Getting Sicker?

**CME Questions**

1. What is the ***most*** common cause of physical disability in Spain?
  - Chronic headache
  - Vision loss
  - Cardiovascular disease
  - Osteoarthritis
2. Which of the following statements regarding the prevalence of painful conditions among patients with disability in the current study of Medicare beneficiaries is ***most*** accurate?
  - The overall prevalence of painful conditions exceeded 75%
  - Headache was the most common painful condition
  - Only one quarter of patients had neck or back pain
  - Less than 20% of all participants reported a painful condition
3. Which of the following statements regarding trends in painful conditions in the current study is ***most*** accurate?
  - Men had a higher rate of painful conditions compared with women
  - Women had a higher rate of painful conditions compared with men
  - Age did not affect the rate of painful conditions
  - Never married was the marital status associated with the highest rates of painful conditions
4. Which of the following variables was ***most*** associated with a higher risk for chronic pain in the current study?
  - Presence of anxiety or depression
  - Sleeping more than 9 hours per day
  - Poor socioeconomic status
  - Nonwhite race

**Evaluation**

## References

[1] Breivik H , Collett B , Ventafridda V , Cohen R , Gallacher D . Survey of chronic pain in Europe: prevalence, impact on daily life, and treatment. Eur J Pain 2006;10(4):287–333. 10.1016/j.ejpain.2005.06.009 16095934

[2] Heiberg T , Kvien TK . Preferences for improved health examined in 1,024 patients with rheumatoid arthritis: pain has highest priority. Arthritis Rheum 2002;47(4):391–7. 10.1002/art.10515 12209485

[3] Fernández-de-Las-Peñas C , Hernandez-Barrera V , Alonso-Blanco C , Palacios-Cena D , Carrasco-Garrido P , Jimenez-Sanchez S , Prevalence of neck and low back pain in community-dwelling adults in Spain: a population-based national study. Spine (Phila Pa 1976) 2011;36(3):E213–9. 10.1097/BRS.0b013e3181d952c2 21079541

[4] Fernández-de-Las-Peñas C , Hernandez-Barrera V , Carrasco-Garrido P , Alonso-Blanco C , Palacios-Cena D , Jimenez-Sanchez S , Population-based study of migraine in Spanish adults: relation to socio-demographic factors, lifestyle and co-morbidity with other conditions. J Headache Pain 2010;11(2):97–104. 10.1007/s10194-009-0176-5 20012124 PMC3452289

[5] Magarolas RG , Clot Razquin G , March Llanes J , Freitas A , Busquets i Bou E , Ruiz Ramos M , Prevalencia de la discapacidad en España por comunidades autónomas: el papel de los factores individuales y del entorno geográfico en su variabilidad. Rev Esp Salud Publica 2009;83(6):821–34. 10.1590/S1135-57272009000600006 20111830

[6] Astin M , Lawton D , Hirst M . The prevalence of pain in a disabled population. Soc Sci Med 1996;42(11):1457–64. 10.1016/0277-9536(95)00253-7 8771628

[7] Valderrama-Gama E , Damian J , Ruigomez A , Martin-Moreno JM . Chronic disease, functional status, and self-ascribed causes of disabilities among non institutionalized older people in Spain. J Gerontol A Biol Sci Med Sci 2002;57(11):M716–21. 10.1093/gerona/57.11.M716 12403799

[8] Thompson DP , Urmston M , Oldham JA , Woby SR . The association between cognitive factors, pain and disability in patients with idiopathic chronic neck pain. Disabil Rehabil 2010;32(21):1758–67. 10.3109/09638281003734342 20350122

[9] Keogh E , McCracken LM , Eccleston C . Gender moderates the association between depression and disability in chronic pain patients. Eur J Pain 2006;10(5):413–22. 10.1016/j.ejpain.2005.05.007 16009583

[10] Pinto-Meza A , Fernandez A , Fullana MA , Haro JM , Palao D , Luciano JV , Impact of mental disorders and chronic physical conditions in health-related quality of life among primary care patients: results from an epidemiological study. Qual Life Res 2009;18(8):1011–8. 10.1007/s11136-009-9522-y 19649768

[11] Ohayon MM . Relationship between chronic painful physical condition and insomnia. J Psychiatr Res 2005;39(2):151–9. 10.1016/j.jpsychires.2004.07.001 15589563

[12] Encuesta sobre discapacidad, autonomía personal y situaciones de dependencia (EDAD). Metodología. Madrid: Instituto Nacional de Estadística (INE); 2010. http://www.ine.es/metodologia/t15/t1530418.pdf. Accessed March 15, 2013.

[13] International classification of functioning, disability and health. Geneva (CH): World Health Organization; 2001.

[14] Nihayah M , Ismarulyusda I , Syarif HL , Zakiah MSN , Baharudin O , Fadzil MH . Sleeping hours and academic achievements: a study among biomedical science students. Procedia Soc Behav Sci 2011;18:617–21. 10.1016/j.sbspro.2011.05.090

[15] Tarsuslu T , Yumin ET , Ozturk A , Yumin M . The relation between health-related quality of life and pain, depression, anxiety, and functional independence in persons with chronic physical disability. Agri 2010;22(1):30–6. 20209412

[16] Leveille SG , Bean J , Ngo L , McMullen W , Guralnik JM . The pathway from musculoskeletal pain to mobility difficulty in older disabled women. Pain 2007;128(1-2):69–77. 10.1016/j.pain.2006.08.031 17055167 PMC2555988

[17] Stubbs D , Krebs E , Bair M , Damush T , Wu J , Sutherland J , Sex differences in pain and pain-related disability among primary care patients with chronic musculoskeletal pain. Pain Med 2010;11(2):232–9. 10.1111/j.1526-4637.2009.00760.x 20002591

[18] Racine M , Tousignant-Laflamme Y , Kloda LA , Dion D , Dupuis G , Choiniere M . A systematic literature review of 10 years of research on sex/gender and pain perception — part 2: do biopsychosocial factors alter pain sensitivity differently in women and men? Pain 2012;153(3):619–35. 10.1016/j.pain.2011.11.026 22236999

[19] Fillingim RB , King CD , Ribeiro-Dasilva MC , Rahim-Williams B , Riley JL 3d . Sex, gender, and pain: a review of recent clinical and experimental findings. J Pain 2009;10(5):447–85. 10.1016/j.jpain.2008.12.001 19411059 PMC2677686

[20] Hirsh AT , Waxenberg LB , Atchison JW , Gremillion HA , Robinson ME . Evidence for sex differences in the relationships of pain, mood, and disability. J Pain 2006;7(8):592–601. 10.1016/j.jpain.2006.02.006 16885016

[21] Agüera L , Failde I , Cervilla JA , Diaz-Fernandez P , Mico JA . Medically unexplained pain complaints are associated with underlying unrecognized mood disorders in primary care. BMC Fam Pract 2010;11:17. 10.1186/1471-2296-11-17 20199657 PMC2837858

[22] Agüera-Ortiz L , Failde I , Mico JA , Cervilla J , Lopez-Ibor JJ . Pain as a symptom of depression: prevalence and clinical correlates in patients attending psychiatric clinics. J Affect Disord 2011;130(1-2):106–12. 10.1016/j.jad.2010.10.022 21055826

[23] Asghari A , Julaeiha S , Godarsi M . Disability and depression in patients with chronic pain: pain or pain-related beliefs? Arch Iran Med 2008;11(3):263–9. 18426316

[24] Von Korff M , Simon G . The relationship between pain and depression. Br J Psychiatry Suppl 1996;30:101–8. 8864155

[25] Bravo L , Mico JA , Rey-Brea R , Perez-Nievas B , Leza JC , Berrocoso E . Depressive-like states heighten the aversion to painful stimuli in a rat model of comorbid chronic pain and depression. Anesthesiology 2012;117(3):613–25. 10.1097/ALN.0b013e3182657b3e 22846678

[26] Scott KM , Von Korff M , Alonso J , Angermeyer MC , Bromet E , Fayyad J , Mental-physical co-morbidity and its relationship with disability: results from the World Mental Health Surveys. Psychol Med 2009;39(1):33–43. 10.1017/S0033291708003188 18366819 PMC2637813

[27] Evans DL , Charney DS , Lewis L , Golden RN , Gorman JM , Krishnan KR , Mood disorders in the medically ill: scientific review and recommendations. Biol Psychiatry 2005;58(3):175–89. 10.1016/j.biopsych.2005.05.001 16084838

[28] Campbell LC , Clauw DJ , Keefe FJ . Persistent pain and depression: a biopsychosocial perspective. Biol Psychiatry 2003;54(3):399–409. 10.1016/S0006-3223(03)00545-6 12893114

[29] Jones A , Zachariae R . Gender, anxiety, and experimental pain sensitivity: an overview. J Am Med Womens Assoc 2002;57(2):91–4. 11991428

[30] Smith MT , Haythornthwaite JA . How do sleep disturbance and chronic pain inter-relate? Insights from the longitudinal and cognitive-behavioral clinical trials literature. Sleep Med Rev 2004;8(2):119–32. 10.1016/S1087-0792(03)00044-3 15033151

